# “I Do not have ADHD When I Drive My Truck” Exploring the Temporal Dynamics of ADHD as a Lived Experience

**DOI:** 10.1007/s11013-025-09910-x

**Published:** 2025-04-29

**Authors:** Gitte Vandborg Rasmussen, Per Hove Thomsen, Sanne Lemcke, Rikke Sand Andersen

**Affiliations:** 1 Department of Anthropology, Aarhus University, Aarhus, Denmark; 2 Centre for Child and Adolescent Psychiatry, Aarhus University, Aarhus, Denmark; 3 Centre for Child and Adolescent Psychiatry, Aarhus University Hospital, Aarhus, Denmark; 4 Department of Public Health, Research Unit of General Practice, University of Southern Denmark, Odense, Denmark

**Keywords:** ADHD, Space, Rhythm, Subjectivity, Imagistic, Natureculture

## Abstract

With this article, we set out to introduce a dynamic and expansive notion of what it means to live with ADHD. Based on ethnographic fieldwork among families living with ADHD in Denmark and inspired by Thomas Fuchs’ *Eigenzeit* [own-time], we forward the notion of “own-time space” as a means of examining the dynamic nature of ADHD. Own-time spaces connect the lived experience of ADHD and time to space. Own-time spaces are situations where the presence or absence of others, and cultural expectations related to timing or tempo enter complex, rhythmic interactions in ways that allow ADHD symptoms to fade into the background. We suggest that own-time spaces are characterized by *space*, *rhythm*, and *imagistic thinking*, and add to our existing knowledge of shielding as a therapeutic effort in ADHD treatment. With own-time space we emphasize that shielding is not just a matter of place or protection from stimuli, but also involves temporal, meaning-making, and relational dimensions. Own-time spaces are dynamic environments where individuals can navigate and negotiate their own rhythms and temporalities and foster a sense of agency and thriving.

## Introduction

Gitte met Kenny in 2011, when she worked in an outpatient ADHD clinic. Kenny has been diagnosed with a severe degree of Attention Deficit Hyperactivity Disorder (ADHD), and since childhood he has been troubled by extreme restlessness, impatience, and an accelerated pace. Kenny had some very difficult periods at school, and because of his unpredictable temper, he would often end up fighting with teachers and other students. During difficult periods, Kenny’s father took Kenny out of school, and for days at a time Kenny would ride with his father in his father’s truck. Kenny has always been obsessed with trucks. He talked about trucks all the time, played truck games on his computer, and dreamt of being a truck driver. When he left his parents’ home, Kenny was introduced to Chris, the mentor provided to him by the social system. Chris’s job was to help Kenny manage his everyday life. He helped him to get up in the morning, taught him to schedule his days, and cook healthy meals, and he coached Kenny in all aspects of everyday life. Gitte met Chris, because he joined Kenny for the clinical follow-ups, and the two of them clearly had a close relationship. When Kenny left his parent’s home to live on his own, Chris became Kenny’s essential lifeline. Kenny was admitted to a special educational program (STU) for people who are unable to follow the standard programs of study in Denmark, and which he was expected to attend daily. He dragged himself through the school, and it was difficult for him to be punctual. Whenever there was an opportunity for an internship or practical work, Kenny succeeded in making arrangements with a truck driver. Kenny and Chris told Gitte that Kenny did well whenever he was in a truck. Behind the wheel of the giant vehicle, Kenny was much calmer, and did not lose his temper in situations where this would usually happen. “It’s weird,” Kenny said one day in the clinic, “but I do not have ADHD when I drive a truck.” He sought Chris’s eyes, and Chris nodded and looked directly at Gitte when he replied, “It’s true. He is capable of so much more when he’s in a truck.”

In this article we take seriously Kenny’s and others’ experiences of ADHD as something that may change or even disappear in specific situations or places, for example, in a truck. The empirical material was gathered in Denmark, and from ethnographic work in families and with adults diagnosed with ADHD^1^, but it speaks to much broader discussions of the nature of ADHD, and anthropological research on neurodiversity, subjectivity, and temporality. We take inspiration from discussions of ADHD as a temporal (Fuchs, 2005, 2013; Goodwin, 2010; Nielsen, 2017, 2020a, 2020b), and embodied (Rasmussen et al., 2024) condition, and suggest that our interlocutors cultivate what we refer to as “own-time spaces” as a means of managing their ADHD. Own-time spaces are spaces or situations where the presence or absence of others, and cultural expectations regarding timing or tempo enter complex, layered interactions with each other, in ways that allow ADHD symptoms to fade to the background. In this article we examine how these spaces work. For Kenny it is simple: His ADHD disappears when he is in a truck, and therefore he returns, time and again. But how does Kenny’s truck-space work to make his ADHD fade away? Seeking out own-time spaces is a helpful way for individuals to responsively and creatively manage their experience of ADHD, because ADHD is not just about being hyperactive. ADHD is also about meaning-making and embodied temporal experience. Own-time spaces reveal the dynamic and fluctuating nature of the lived experience of ADHD, thereby adding to the research that suggests that ADHD is a nature–culture, temporal, and dynamic condition (Brinkmann, 2024; Nielsen, 2017, 2020a, 2020b; Rasmussen & Meinert, 2019; Rasmussen et al., 2024).

### Diversifying ADHD

Globally, the number of individuals with ADHD is on the rise, and ADHD occurs unevenly, both locally and globally. A Danish study from 2015 showed a variation in the incidence of ADHD, from 0 to 2.87%, in the administrative municipalities (Madsen et al., 2015). Worldwide, the prevalence of ADHD has varied between 0.1% (in Iraq) and 8.1% (in the USA) (2017 figures, based on children and adolescents).^2^ In Denmark, the rise of ADHD, and the geographic variation in the prevalence^3^ is poorly understood, but research on ADHD is expanding. This is not the place to review all the literature, but as noted by scholars such as Svend Brinkmann and Thomas Fuchs, there is a growing body of literature which conceptually seeks to integrate neurobiological perspectives and culture or ecological perspectives. Briefly, the neurobiological perspectives address ADHD as a disorder of the brain (primarily) caused by low levels of neurotransmitters in the synapses. This is the dominant explanation in the field of psychiatry, and the basis of psychoeducational training, although in combination with an evolving environmental approach (Barkley, 2020). Social sciences scholars often take a critical stance on what they term the essentialist or naturalist understandings of the neurobiological perspectives, and argue that the variation and rise in ADHD (and mental illness, more broadly) is due to a complex interplay of environmental factors (Conrad, 2007; Grinker, 2009; Rose, 2007), or what we may think of as subject or neurodiversity (Rose, 2007; Rapp, 2016; Christensen, 2021; Glavind, 2022; Stokker-Jensen, 2017).

The British sociologist Nicolas Rose and others have raised markedly critique of the neuro-dominance in the biomedical model, arguing that it encourages individuals to perceive themselves primarily as chemical entities and to actively seek diagnoses (Rose, 2007). Also, the psychiatric paradigm’s growing emphasis on the brain has been criticized as perpetuating a reductionist view of human beings (Fuchs, 2018), and of ignoring the fact that the rise in ADHD diagnoses may also be attributed to the broader medicalization of society, including the commercialization of Ritalin (Conrad, 2007).

Per Hove Thomsen (2015), a Danish psychiatrist, suggests that the emergence of diagnostic manuals such as DSMV and ICD10 automatically add diagnoses, and the de-tabooing of living with mental illness has led more people to seek diagnoses of their psychiatric conditions. In the field of social science studies, the rapid rise in diagnoses, for example autism, has been described as an awareness epidemic (Grinker, 2009), and scholars have demonstrated that living close to other people who have been diagnosed increases the probability of being diagnosed with a similar condition, because knowledge of, and attention to symptoms and treatment is communicated among neighbors and in social networks (Seeberg and Christensen, 2017). In our empirical material, this also holds true for ADHD, as we see how family members and friends inspire one another to seek diagnoses because they recognize their relatives’ ADHD symptoms in themselves, and thus, diagnoses spread through the families. Furthermore, in Denmark, a(n ADHD) diagnosis gives access to benefits from social services, which may also contribute to the rise in ADHD diagnoses (Nielsen, 2020b, 2021).

There are several perspectives on what ADHD is, and why it varies with place and time, and researchers identify different factors that affect the rise of ADHD. Brinkmann (2024) suggests that instead of consider nature and culture as separate domains, we should *“*develop a “naturecultural” approach to psychopathology that avoids mentalism” (ibid: 1). In this article, we were inspired by Brinkmann’s work, and approach ADHD as a dynamic, natureculture condition, which emphasizes the fluidity of ADHD, both as a phenomenon (what ADHD is) and as lived experience (how it is lived). Approaching ADHD as a natureculture phenomenon we thus attempt to include both nature/biological and cultural/social perspectives on ADHD, which supports our line of argumentation and helps us unfold complexities of ADHD experiences. We do this while adding to the developing literature on ADHD and time from both social science and neuropsychiatry (Barkley, 2005; Barkley et al., 2001; Carelli & Wiberg, 2012; Goodwin, 2010; Nielsen, 2017, 2020a, 2020b; Rasmussen & Meinert, 2019; Rasmussen et al., 2024).

### ADHD as a Lived, Temporal Experience

Kenny’s greatest challenges are restlessness, a lack of focus, and a short temper. It is impossible to have a long conversation with Kenny. He gets up from his chair, and after a while, he also leaves the room and the conversation. Often, he is restless, and sometimes this restlessness leads to irritability. When he was younger, he suggested that conversations in the clinic should be no longer than 10 min (instead of the scheduled 30 minutes). He would start a timer, and prompt Gitte throughout the conversation to stick to the time. His legs trembled, and his fingers tapped the table. If Gitte was too slow, Kenny would loose his temper. As a child, Kenny experienced serious side-effects from his ADHD medication, but as an adult, he tolerates his medication, and his body is more at ease. Yet, trees do not grow into the sky, and even though Kenny is now better off because of his medication, holding a long conversation is still a huge challenge.

Having trouble with tempo, and sometimes temper, like Kenny’s impatience and short fuse, are core symptoms of ADHD often reported in the literature (Barkley, 2005; Goodwin, 2010; Nielsen, 2017, 2020a, 2020b; Rasmussen & Meinert, 2019). *Hyperactivity* is being too fast compared to surroundings, *impulsivity* is failure to think before (re-)acting or speaking, and *attention deficit* is the inability to focus on finishing a task or activity. Psychiatrists Bilenberg and Hjerrild (2024, web) describe how ADHD often causes additional chronobiological disturbances that lead to forgetting appointments, a distorted sense of time, difficulty with transitions from one state to another (e.g., sleep and waking, not focused to hyper-focused), disturbed sleep patterns, and hormonal sensitivity. Further, anthropologists described how a hyperactive body tempo can mutually affect family members, for example, and sometimes create a hectic atmosphere (Rasmussen et al., 2024).

Deficits in executive functions related to planning and scheduling events are also described as significant problems related to ADHD. Russell Barkley, an influential psychologist, suggests that living with ADHD is being *time-blind* (2005). In clinical trials Barkley and colleagues have shown that people diagnosed with ADHD have a faster internal rate than people in a control group (e.g., Barkley et al., 2001), and they argue that individuals with ADHD “live in the moment”, which makes them more aware of, and sensitive to, the present, than aware of (planning for) tomorrow (Barkley, 2020:162). Barkley’s work on time and ADHD has been an important source of inspiration to us. We wish, however, to add to his work by bringing more nuance to the ways in which time and ADHD are brought together in conceptual representations such as “time deficits” and “time-blindness”, and we do this by adding a critical phenomenological account of lived experience (cf. Fuchs, 2017, 2018; Mattingly, 2014, 2019). Our interlocutors describe themselves as being too fast or too slow, compared to their surroundings. This becomes apparent when someone’s bodily pace clashes with social time. For example, in the case of 12-year-old Rasmus, who is the fastest player on his football team, or in the case of Kenny, who speeds up conversations, and who was chronically late for school as a child. The ways in which individuals who live with ADHD experience time as embodied “haste” or desynchronization (Nielsen, 2017), and how they respond to societal temporal regimes (Carceral & Flaherty, 2021), we suggest, are central to our understanding of ADHD. But relations between time and ADHD should not be considered only through the lens of deficits. Our interlocutors engage with their experience of time and temporal regimes through timework^4^ (Nielsen, 2020a, 2020b; Rasmussen et al., 2024), and they cultivate own-time spaces in an often creative and responsive manner. We suggest that this has implications for the way we understand relations between time and ADHD as well as existing therapeutic efforts, such as shielding.

In Denmark, shielding is often employed in special classes and in schools for children with ADHD and other psychiatric conditions. Dividers shield individual workstations to prevent students from being disturbed, and thereby improve their concentration, and the physical environment is sometimes designed with small, shielded places or “oases” that allow for withdrawal. In special schools, children with ADHD often work behind screens, to deliberately shield them from the movements of other pupils, the scribbling of pencils, or noisy keyboards that might lead to distraction and loss of attention (Olsen and Christensen, 2013, web). These implications extend to societal structures and temporal regimes from which individuals with ADHD are shielded. The way contemporary society is organized affects how individuals with ADHD and other forms of neurodiversity thrive.

### Relationality, Temporality, and Space

In his work on mental illness, Fuchs discusses “the brainification” that the neurobiological paradigm has brought to the psychiatry. According to Fuchs, the brain is a relational organ that includes the *Umwelt* (2018: preface v): “A mediating organ of potentiality” (ibid., 289) rather than “the creator of the world” (ibid., 291). We are not our brains, Fuchs argues, but living human beings with brains:

Human persons become at one with themselves, not in a mental or neuronal inner world, but in their bodily and inter-bodily “being-in-the-world” and “acting-in-the-world” (ibid., 291).

When Fuchs states that the brain involves the *Umwelt*, he emphasizes that neither the brain nor an individuals’ perception of their environment are fixed, stable, or separable, objective phenomena. Rather Umwelt *is* the individual, subjective world—or lived experience—that is shaped by sensory and temporal experiences and personal engagement with the environment. Pointing to the brain as a relational organ, Fuchs emphasizes that perception is not a passive process of receiving sensory input but an active engagement with the world (2018: preface V), and the study and analysis of psychopathology and time experiences should always take an inter-subjective stance: “Individual lives and experiences of temporality cannot be grasped without implicit or explicit reference to the contemporaneous lives of others” (2013, p. 76).

In personal conversations with Fuchs, regarding our work, he introduced the notion of *Eigenzeit* [own-time] as a broader concept than chronobiology. While chronobiology concerns the influence from biological rhythms and cycles (sleep-wake, hormonal and seasonal changes) on human behavior and physiology, *Eigenzeit* refers to the notion of being and living a body, and to embodied, subjective time. Subjective time, or embodiment and temporality, combine in bodily processes such as arousal, relaxation, or motivation, which manifest in heart or breathing rates (2020, p. 14), and also in the recurrent dynamic of “cycles of lack and fulfillment, expenditure and regeneration, waking and sleeping” (2020, p. 15). To our knowledge, the concept of Eigenzeit has not been developed elsewhere.

In this article, Kenny’s case and other illustrative examples serve to broaden Fuchs’s concept of *Eigenzeit*, to include attention to the broader cultural context of human experience, such as the complex interplay between individual experiences, meaning-making, and relational dynamics (Danely, 2022). On a general level, we believe that our interlocutors cultivate spaces of their own, because human subjects are both temporal beings (Flaherty, 2011; Fuchs, 2017; Lefebvre, 2022; Rosa, 2014, 2019) and inter-subjective beings (Meinert, 2022; Osawa De-Silva, 2021). Humans are far from defined by “what goes on in the brain” or in the interior of an impervious entity, but are “porous” (Fuchs, 2017; Osawa de-Silva, 2021). Subjective experience (such as the lived experience of ADHD) is the product of the interplay of biology and environment, or nature and culture, including social and political environments (Osawa de-Silva, 2021, p. 30). On a biological level, human subjects are flesh and blood. We can experience sensations such as “the blood rising” or the heart pounding due to unrest. Some biological processes unfold in close connection with the environment, for example, when Kenny becomes unruly and restless during long conversations, and his legs start to tremble, and his fingers tap the table.

On a social level, we take inspiration from anthropologist, Osawa de-Silva, who describe subjectivity as “a process of differentiation that establishes a divide—albeit a porous one—between what is ‘self’ and what is ‘not-self,’ or ‘other’” (2021, p. 20). This means that it is through sharing a world with others that subjective experience is realized (Fuchs, 2013; Jackson, 2018). Subjective experience is thus Janus-faced, or inter-subjective, in the sense that a subject is always simultaneously oriented toward the collective, and the self (Osawa de-Silva, 2021; Fuchs, 2013; Rosa, 2014, 2019). In other words, human subjects are both self and other—alone and not alone. This definition of subjectivity helps us understand how own-time spaces work, namely because the subject is a Janus-faced entity—always alone—or a self and always a “we”. When Kenny is unruly, he may try to communicate it, and others may see it, and respond. Although Kenny may respond to others’ responses, others can never fully experience what Kenny does. Also, Kenny’s experiences of himself and his world, “are continually bound up in an ebb and flow” of reflexive and reciprocal interaction (Jackson, 2018, p. 185). Kenny is a self-conscious and reflexive being, who responds to the stimuli from his truck or from other fellow human beings. We suggest that this two-sidedness of subjectivity, and the fact that it is both social and biological, adds to the lived, temporal experience of ADHD. Human needs for “own-time spaces” are the result of this dynamic openness to the world.

We often think of time as something that primarily exists outside the human subject; as structures that occur in sequences, and which can be measured in units such as seconds, minutes, and hours, or as more abstract phenomena that “can pass,” and as something of which humans can vaguely perceive. But time is also part of human subjectivity (Flaherty, 2011; Fuchs, 2017). We live through time and experience pasts, presents, and futures. Moreover, the human experience of time is “thick”: when we experience ‘now’, we are also always aware of and sense both a past and a future (Husserl, 1964: 176, see also Fuchs, 2017, and Flaherty, 2011). For one thing, humans can do time work: They can reflect on time, and consciously prolong it, allocate it, or “make time fly by” (Flaherty, 2011). But the human experience of time is also deeply connected to subjective experience, both as bodily rhythms (racing thoughts, feeling the heart pound) to temporality, and the social experience of time (Fuchs, 2017; Lefebvre, 2022). For example, the Danish anthropologist Mikka Nielsen, who is inspired from Fuchs´ work on temporality and psychopathology (Fuchs, 2005, 2013) suggests that ADHD is a state of desynchronization, and that they are “out of sync” with their surroundings (Nielsen, 2017, 2020a, 2020b). Nielsen’s work among adult individuals living with ADHD in Denmark shows how ADHD becomes particularly apparent in the clashes between internal and external rhythms, and how her interlocutors use timework strategies to modulate their experience of ADHD.

We add to the foregoing by showing that our interlocutors do not only carry out time work (2011), but also make use of space to cultivate situations of thriving. In own-time spaces, we see how ADHD symptoms may vanish, not just because of rhythm and synchronization, but because of an overall improved thriving. Own-time spaces are spaces where the experience of ADHD—the sense of restlessness, hyperactivity—fade or dissolve, and even sometimes vanish into thin air. We suggest that own-time spaces are places or situations where the presence or absence of others, and cultural expectations regarding time or tempo, enter a complex, rhythmic interaction with each other in ways that allow the subjective experience of ADHD to fade to the background. More specifically we suggest that own-time spaces are characterized or layered by *space*, *rhythm*, and *imagistic thinking*, which have a direct effect on the embodied experience of ADHD. Yet, this concept adds to our understanding of what it is like to live with ADHD, and it underscores the importance of social interaction, and the significance of meaning for temporal, embodied experience.

## Ethnographic Setting

As we have indicated, Kenny has access to various kinds of social and healthcare support. The Danish welfare system provides healthcare and social services that are funded primarily by taxes, and thereby mostly free of charge. This tax-supported system aims to guarantee equal access to social rights and healthcare for all residents in Denmark (Laursen, 2023). Although an expanding social inequality has been described, most people live fairly secure lives, and can count on social benefits when they fall ill, or are unable to work. ADHD assessment is handled by the psychiatric system and, in the case of children, in close collaboration with the PPR [Pædagogisk Psykologisk Rådgivning], which is a pedagogical and psychological team working closely together with schools and teachers. For children, social pedagogical/educational support such as structure, shielding, and psychoeducational training (information about ADHD, coaching, or cognitive therapy) are recommended as the first choice of treatment, and medication as the second choice. For adults, ADHD medication is recommended as the first choice of treatment. Medical treatment is handled by either a general practitioner or a psychiatrist, or is provided by a public ADHD clinic as part of psychiatric care. Psychoeducational training and social pedagogical support are offered via social services, and are often provided by a mentor, as in Kenny’s case, where Chris is his mentor. Apart from mentors, many interlocutors work a reduced number of hours, and receive compensation, benefits frequently provided to adults with ADHD. Financial reimbursement for medication is also common. Opportunities for social services vary among municipalities. In Kenny’s case, this meant that when he moved in with his girlfriend Anne in another municipality, he and Chris ways parted because Chris works in Kenny’s home municipality. The Danish ADHD association offers seminars, conferences, and so on; here, payment is required, but is sometimes repaid by the social system. When it comes to children, the PPR and school teachers are closely involved in therapeutic efforts. As we mentioned, a diagnosis is often needed to get access to benefits from the state (Nielsen, 2021).

## Exploring ADHD

When Gitte began her fieldwork in 2020, she was familiar with ADHD because of her clinical background in an ADHD clinic, and she was also familiar with some of the interlocutors whom she knew as former patients, or former interlocutors from a previous research project. Yet, there were still new interlocutors added in 2020. Gitte’s fieldwork was carried out over 2 years (2020–2021), and consisted of roughly 3 parts: (1) the main fieldwork, in which 8 Danish families were followed, and included participant observation (O´Reilly 2012) and semi-structured interviews (Bernard, 2006); (2) during the Covid-19 lockdown, 21 adult interlocutors were added to the cohort, and semi-structured were carried out online; and (3) two psychoeducational groups for women were followed for 20 weeks (one meeting per week, online or IRL).

All the interlocutors were diagnosed with ADHD, and according to the families, at least one family member had received the diagnosis. Many people living with ADHD also have comorbid psychiatric conditions (Gillberg et al., 2004). This was also the case with the interlocutors in this study, where diagnoses such as autism, OCD, and anxiety were the most common, after ADHD. Gitte followed some of the interlocutors for several years, and she saw how diagnoses frequently changed as ongoing diagnostics and assessments continued. Therefore, it is hard to account for comorbidity when doing long-term research. Also, since ADHD often presents with comorbid psychiatric conditions, attending to the lived experience with ADHD means attending to the intricate conditions of comorbidity. What ties together the interlocutors’ narratives and experiences is that they all refer to ADHD as their main diagnosis.

When Gitte introduced the aims of her research to the interlocutors—her interest in learning how they experience time (being on time, the flow of time) and temporality (body-time, rhythms, tempo) in everyday life with ADHD—no interlocutors questioned the logic of combining these topics. Instead, it was welcomed, and prompted reflection on punctuality, pace, or unrest. Anders, for example said, “being punctual is absolutely the most difficult thing to cope with, because I have no sense of time,” and Sofie described the recurring experience of a lack of time in high school: “if I only had more time in school, I could easily manage my tasks.”

Studying, observing, and looking for time in families may be ephemeral. In addition to discussing interlocutors’ time experiences and the kinds of time work in which they engaged (Flaherty, 2011), Gitte paid attention to their surroundings (e.g., corners designed in the homes for shielding/withdrawal/privacy, schedules and calendars, timers, and other time-regulating objects and spaces). By sensing and listening carefully to the atmosphere (Stevenson, 2014) in the homes that sometimes became hectic because of a rapid pace or slowed because of extreme fatigue, Gitte could attune to the subtle shifts in the environment. Drawing on openness and curiosity as a methodological tool (ibid.) allowed for moments of pause and reflection. These changes in atmosphere and tempo sometimes affected the anthropologist’s body and mind, as described by Nielsen (2017).

We base our analysis on all the ethnographic material mentioned above, but throughout the article, we will return to Kenny as the main figure, because his case has many layers, as Gitte has known Kenny and his family since 2010.

### Spaces: Trucks and Flats

Like Kenny, other interlocutors use space to balance proximity and distance from other people, and from situations that they find disturbing, or that cause them unrest. Apart from his truck, Kenny also has his own corner in the living room, which allows him to find calm and a bit of privacy, and which gives him the opportunity to do what he wants to do. “In my truck I decide for myself,” Kenny says. His time is flexible, in terms of eating or listening to music whenever he wants. He can make a stop and buy fast food from the petrol station, or listen to loud music on the radio without disturbing, or being disturbed by, anyone. At night, Kenny’s girlfriend Anne stays awake so she can talk to Kenny on the phone. They both light up when they talk about this in an interview, and they both obviously enjoy these nightly conversations. Spending time “together apart” (e.g., on the phone and in the truck) and “close at a distance” is ideal for Kenny and Anne. They both enjoy living together only during the weekends, and as long as Kenny has his own corner in the living room in which he can withdraw.

Sometimes, spaces are created by interlocutors or allocated as an agentic strategy like timework (Flaherty, 2011). Hence, they may also be introduced by others, such as Kenny’s father, who introduced Kenny to his truck, or they may be discovered by chance, as Anders and Merete did during the Covid-19 lockdowns, when they discovered how much they liked being at home in their own apartments. Merete, who was diagnosed with ADHD, lives alone, and experienced great relief and throve because she worked from home and was freed from frantic mornings and other stressors. The same held for Anders, who has ADHD and Asperger’s Syndrome, and is particularly vulnerable to stimuli such as wind, sound and light. He enjoyed staying in his apartment, and not having to deal with disturbing sensations or affective disturbances from the world outside his flat. Being home alone or alone in a truck allowed Kenny, Anders, and Merete to carve out and create a time and “a space of individuality from which to be social” (Meinert, 2022, p. 139).

On a general level, we believe that our interlocutors cultivate spaces of their own, because, as we discussed, human subjects are (inter)subjective and temporal beings (Fuchs, 2017; Husserl, 1964; Lefebvre, 2022; Meinert, 2022). Our sense of self and our human ability to experience the world are deeply influenced by interactions with others (Fuchs, 2017, 2018). “The dynamic encounter between two (or more) lived bodies typically colors space with a familiar sense and open the door to an array of social experiences” (Bader et al., 2022, p. 129). Human experience of space is inherently formed through interactions with other human bodies, or what Laurent Berlant has called “the inconvenience of other people” (2023, p. 2). When Berlant writes about spaces of individuality or the inconvenience of other people, it should not necessarily be understood negatively. Instead, it refers to the temporal, affective, or embodied “sense of friction” (ibid.) of being in proximity to others, of having to adapt to, or take into consideration a glance, the movement or the unrest of the other, or of having to adapt to the desires or forms of social exchange that come with the proximity of others.

Meinert (2022) draws on fieldwork among the Ik people of Uganda, and presents the concept of *fencework* to describe how fences made of wooden sticks are built around family huts in the village, and establish the possibility of privacy within the families, but also to invite other people whenever they are needed. Fence-work is carried out for the social purpose of balancing proximity and distance to other people. Berlant and Meinert write from two different cultural contexts (the US and Africa), and not about individuals who live with ADHD. However, we believe that the mechanisms of space and proximity are even more important to individuals who live with ADHD. Many individuals who live with ADHD are sensitive to stimuli such as sound, smell, and light in their surroundings (Blum et al., 2008), and may find intersubjective, temporal coordination and timing difficult. From a neurobiological point of view, this is caused by low levels of dopamine in the synapses, and reduced function of the pre-frontal cortex, which filters out environmental stimuli. Therefore, in popular terms, individuals with ADHD are said to be “living without a filter,” and tend to become overstimulated^5^ and stressed. Here, we add to neurobiological explanations, by pointing out that individuals with ADHD may be extra sensitive to time, rather than being time-blind.

In Denmark and elsewhere, shielding is a widely recognized therapeutic effort to regulate ADHD symptoms^6^. We see how interlocutors use headphones to shield themselves from noise, wear a hat or cap some days, avoid shopping during rush hour or in huge supermarkets, or create private spaces in their homes, such as Kenny’s corner of the living room. Kenny’s truck-space acts as a kind of shield for him, by protecting him from stimuli. But apart from this, it allows him to work on time: He can decide when to take a break, and whether he wants to help unload a cargo or stay in his truck and communicate only over the walkie-talkie. Also, Kenny appreciates the solitude and the bodily ease and calm the truck provides. Sometimes, he just needs to be on his own, and the solitude of his truck allows him to “work out some kind of ‘positioning’ that reconciles” his needs and others’ needs (Jackson, 2018, p. 186). However, own-time spaces differ from shielding: They do not merely establish boundaries, but are part of a broader cultural context of meaning-making and relationality. Next, we will focus on how various dimensions of images and rhythm contribute to the creation of own-time spaces.

### Imagistic Thinking: Yolks and Space Capsules

Interlocutors often use evocative images such as “jumping popcorn-thoughts,” “feeling hyperactive inside,” “having ants running over the body,” or being a “rapid speaker” when describing how they experience ADHD. In her writings, Mikka Nielsen also pays attention to her ADHD interlocutors unique vocabulary, and how they “paint pictures of ADHD.” For example, Karen, Nielsen’s interlocutor, notes, “I am hyper on my speech.” In our data, we see how interlocutors create new words or idioms, perhaps because the Danish language is not rich enough, or because they sometimes stumble over their words due to their racing thoughts. Our empirical material is full of creative, humorous, and profoundly serious examples of interlocutors “speaking ADHD-ish” (Nielsen, 2020b, p. 2), or, to borrow from Lisa Stevenson, what we describe as “imagistic thinking” (Stevenson, 2014, p. 10). Kenny’s repeated insistence on returning trucks, be it in his drawings, computer games, stories, or dreams, is an example of *imagistic thinking*. By presenting the rhetorical images evoked by our interlocutors we show how “imagistic [..] modes of knowing allow us to be faithful to a whole range of contradictory experiences” (ibid., 10). Kenny’s experience of living with a severe degree of ADHD, while insisting that he does not have ADHD when he drives his truck, presents such a contradiction, as we will see next; so does his tattoo.


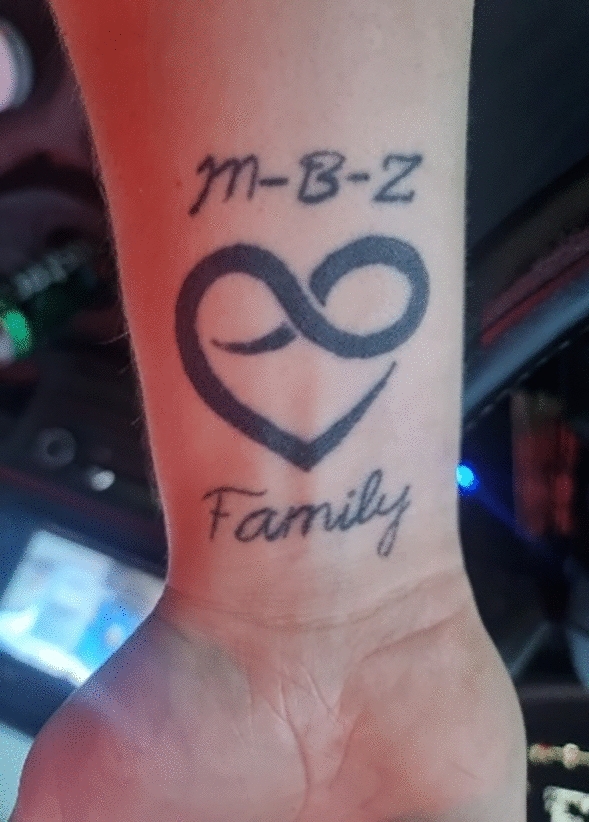


In January 2020, Gitte was interviewing Kenny in Anne’s apartment. Kenny had finally passed the driving test, and had become a truck driver. On this day, Kenny welcomed Gitte at the doorstep, where the faint smell of his last cigarette was still in the air. In the narrow hallway, Kenny’s work clothes—an orange coverall—hung from a hook. Kenny gestured with his arm, and led Gitte into the neat and clean living room. Kenny had his own corner of the room, where his computer and gaming chair were shielded by a tall bookcase, which gave him a bit of privacy and rest in the tiny home.

While Anne was in the kitchen boiling water for instant coffee, Kenny invited Gitte to his private corner. Kenny guided Gitte through a virtual tour on Google Maps, and showed her every detail of his night drive in his truck. He was eager, and zoomed in and out at every warehouse, and explained where and how the loading was done, and suddenly exclaimed, “It is still the same, I do not have ADHD when I drive my truck.” Kenny’s leg was trembling and he moved slightly back and forth in his excitement.

The coffee was made, and Anne invited for a cup at the small table. Kenny turned off the computer, but hesitated, slowed down, and seated himself again. “I have something to show to you,” Kenny said with a new calm in his voice. He pulled up his sleeve and displayed a tattoo. It was a heart with the initials of Kenny’s father, mother, and sister above it, and the word “Family” under it. The heart was drawn as a continuous line, the number 8 lying down, like the infinity symbol, with the letter V as continuation of the 8. These signs are symbolic of his truck, which has a V8 engine with 8 cylinders placed like a V, Kenny explains. Knowing Kenny’s life story, it is striking how inventive his tattoo is. The truck has been crucial in Kenny’s life, and has served as a motivational drive through his studies and work life. However, bearing in mind Kenny’s tattoo, and carefully considering his life story, it becomes clear that more is in question than the truck-space that serves as a shield and mechanical block to stimuli and disturbing rhythms. Kenny’s tattoo is an image of the truck itself, and initials of his immediate family embrace his tattoo heart. Images, coined by Foucault in Stevenson, “express without formulating” (2014, p. 12). Here, Stevenson does not discuss the concrete expression of an image, but of the image that is left like a sensation that moves or settles in the subject. Even after stories are attempted to be told, images stay, they are able to “hold” us, beyond formulation (ibid., 12). This is exactly what Kenny’s tattoo did, when Kenny slowed down and reached out to Gitte to show her his tattoo, and the image of the tattoo is a testimony that adds multiple layers that tell of Kenny’s love of his truck, and why it comforts him. Anne, and Kenny’s father and mother, whose initials are part of the tattoo, add relational and temporal meaning to the space of the truck. The truck is not just a vehicle, but a vessel full of connections, and an image of the intricate blend of lived time and kinship, which transforms the truck into Kenny’s own-time space.

The telling significance of images is also present in cases other than Kenny’s, where own-time spaces are described in imagistic terms. Merete, whom we already introduced, had been diagnosed with ADHD for 12 years, and she stated that her ADHD symptoms did not bother her much during the first Covid-19 lockdown. Freed from time-pressure and the need to rush out the door in the morning, and free of having to be in specific places at specific times throughout the workday, Merete stated, ”I feel like the yolk in an egg.” To feel like the yolk in an egg [*blommen i et æg*] is a Danish expression that means that you live a privileged, protected, and safe life. The interview was carried out online, and Gitte still recalls Merete’s smiling face on the screen: She wore a yellow dress, and against the backdrop of the white walls, she was literally the image of “a yolk in an egg.”

Anders lives alone, and was very pleased to work from home during the Covid-19 lock down. As a child, Anders dreamt of having his own space capsule: A closed space with a control panel with buttons for regulating stimuli such as heat, noise, wind, light, and so on. Anders did not become an astronaut; instead, he became an astrophysicist, and working from home during the lockdown helped him to thrive (cf. Weinstein et al., 2021). His ADHD symptoms decreased markedly: Alone in his flat, Anders did not need to be punctual, which he always found difficult, he decided for himself if he wanted to leave the apartment, and he was able to control incoming stimuli and sensations. In an online interview, Anders sat in the middle of his living room behind his desk, smilingly and comfortably flapping his arms, as he exclaimed: “Look, I ended up getting my very own space capsule after all.”

Anders’s experiences are similar to Kenny’s. Like Kenny’s truck, the enclosed space of his flat helped him regulate his experience of ADHD. Yet, if we view the truck/tattoo and the flat/capsule as examples of imagistic thinking: We learn that the truck and the flat represent deeper meanings tied to their temporal, lived experiences, dreams, and relationships. Like human experience, images may be understood as temporal phenomena linked to the flow of time (Husserl, 1964) and manifestations of past, present, and future. Images of trucks and space-capsules invites both the past and the present into their own-time-spaces.

Attending to the imagistic thinking of Anders and Kenny thus implies that their experience of ADHD is influenced not only by the physical shielding these spaces provide, but also by the temporalities and meanings attached to the flat and the truck. The space capsule was a boyhood dream come true, just as the truck was for Kenny, as it connected him—in time—to his father and provided a meaningful way of living. In the truck and the flat, Kenny and Anders were synchronized with the surrounding world (Nielsen, 2020a), in the sense that they became less unruly, and their need to do time work, in terms of adhering to society’s calendar regimes, was reduced. But this synchronization relied heavily on the fact that they were personal spaces, or own-time spaces.

## Rhythm

Own-time spaces are also rhythmic. Not only do Kenny’s truck and Anders’ flat offer an embodied, temporal rhythm to adapt to, but to some extent, own-time spaces “rhythm” whole lives for some time. When Kenny is deeply seated in his huge, humming truck, and his eyes are fixed on the endless road’s horizon, he finds calm. Driving an enormous truck for many hours, sometimes with a trailer, requires concentration and focus. As Kenny demonstrated during the virtual guided tour he presented in his living room, driving a truck is a matter of many practicalities, routines, and repetitions: constant maneuvering in traffic, shifting gears, checking the mirrors, braking, loading, unloading, starting again, checking the mirrors, stepping on the accelerator, back on the road.

Repetition establishes a form of rhythm that is described as helpful for people suffering from trauma, and predictability and routines are known to ease ADHD symptoms (Williams & Meinert, 2020; Barkley, 2020, pp. 252–253). The predictability of routine work, such as that of a truck driver, may add to the experience of calmed bodily rhythms, and the deep, monotonous rhythm of the truck engine may rhythm (Lefebvre, 2022, p. 78) or calibrate (Nielsen, 2017) Kenny’s hyperactivity and restlessness. The physical space of Kenny’s truck has certainly served as a kind of shield against stimuli, and also from disturbing external rhythms that threaten to disturb or muddle Kenny’s own rhythms. The truck, it seems, rhythms Kenny, and the hyperactivity, unrest, and irritability that embody his ADHD disappear.

Daily visits to a livery stable with her daughter Mille helped Pia to manage her racing thoughts and what she described as “the fast pace of her body.” Pia was diagnosed with ADHD as an adult; Mille has not yet been diagnosed, but Pia believes that Mille has ADHD too, because she recognizes herself in Mille’s quick tempo and temper. Mille adores horses in all aspects of her 7-year-old life, and Pia recalls her own childhood excitement about horses. Following what Pia terms a “horrific period,” because of her mother’s serious illness, Mille’s severe problems in school, and Pia’s increased ADHD symptoms and anxiety, Pia and Mille agreed to look after a friend’s horses on a daily basis at a stable nearby. Gitte went with Pia and Mille to the stable, and this is an excerpt from her field notes, which begin in Pia’s car:

Mille’s voice is eager: “Mom, I’ll mix the food and you fix the hay.” We are three people stuffed into Pia’s tiny car on the way to the stable. As always, Mille’s good vibes spread, the atmosphere is great, and everyone is excited. At the stable, we are met by four big chestnut horses. Speaking for myself, I am not that confident with horses, and I am quite astonished as I watch tiny Mille bumbling among the horses, mixing the food, handling the heavy buckets, and pushing the huge animals in a mature way to make the horses move. Pia fixes the hay, and like Mille, she moves fearlessly, decisively, and, in my eyes, very close to the horses. Mille now shovels horse shit while I carry buckets of water and sweep the floor. My arms are heavy, this is hard work! I sense both Pia and Mille’s strength. They work closely together, and we rarely speak. The stable is swept in this weird, wordless silence, weird because the three of us are usually loud and very talkative when we are together. Mille has been taught all this by Pia, I reflect, by copying her way of touching the horses and moving around in the stable, and I sense the peace between Pia, Mille, and myself, and note the calm of the horses.

Some hours later, Gitte is drinking coffee in Pia’s kitchen, and Pia is explaining how visiting the horses with Mille has affected both her and Mille’s restlessness positively. Pia loves to spend time with Mille, but at home, they sometimes end up arguing or starring into their own screens. In the stables, they must work closely together and focus strictly on the horses. Pia explains how this eases their hyperactive bodies, while also calming their conversations and reducing the friction (Berlant, 2022, p. 2) that sometimes emerges when they spend time together: “It is because you can’t be wild among horses, then the horses will go wild, too... so, this grounding we both get from working with the horses makes quite a difference.” As in Kenny’s case, the physical, rhythmic, and repetitive work, day after day, and being close to the huge animals, serves as a kind of insistent metronome for both Pia and Mille’s body tempi. If we add to this their shared passion for horses, we see how time spent at the stables rhythms both Pia and Mille’s lives.

Lefebvre distinguishes between cyclical rhythms (natural, repetitive cycles, e.g., days, seasons, and biological rhythms) and linear rhythms (mechanical, industrial, and social rhythms). He says, that the body is polyrhythmic, and rhythms may either synchronize or conflict with each other. In arrhythmia, “rhythms break apart” (2022, p. 77), or fall into disorder with each other, which Lefebvre describes as a pathological situation. Lefebvre’s concept of arrhythmia aligns in many ways with Fuchs’s concept of desynchronization (Fuchs, 2005), and the way Nielsen has applied the idea of desynchronization to everyday life with ADHD in Denmark. As introduced, Nielsen points to ADHD becoming visible in rhythm clashes, and emphasizes that ADHD is *a desynchronized way of being in the world* (2017). Nielsen notes how her ADHD interlocutors use medication or movement to calibrate intense restlessness in the body and mind, and Lefebvre notes that individuals are rhythmed and disciplined by political power, through the manipulation of time and time-tables (2022, p. 78).

In our empirical material, we observe how interlocutors are sometimes rhythmed by horses, nature, or trucks. We suggest that rhythm is central to understanding how own-time spaces work. Rhythm manifests as embodied repetition and the experience of being “in-sync,” and the routines and repetitions of bodily work add appreciated forms of predictability to situations. However, we also suggest that rhythm extends beyond a specific situation. Whole lives may be rhythmed, in the sense that own-time spaces provide a sense of agentic maneuvering of temporal regimes (Carceral & Flaherty, 2021), which offers a break from difficult-to-manage lived circumstances, and is a means of recalibrating social relations. In this sense, own-time spaces are not only spaces with particular rhythms, but may also rhythm entire lives.

## ADHD, temporality, and thriving

The cultivation of own-time spaces—understood as spaces characterized or layered by *space*, *rhythm*, and *imagistic thinking*—lays the foundation for meaningful thriving or living well with ADHD. Attention to own-time spaces, and the specific practices, relational sensitivity (Baden et al., 2022; Berlant, 2022), and time work (Flaherty, 2011) involved in cultivating these spaces, teach us how humans experiment (and often struggle) with building a meaningful life with ADHD in a society that values calendar-structured lives and timeliness.

Other researchers have written about how human subjects who live in troubled times seek out spaces or environments that suit them and help them thrive. Osawa de-Silva (2021) distinguishes between the Japanese concepts of *ikigai* and *ibasho* when she writes about loneliness and suicide in Japan. Ikigai relates to one’s purpose in life and meaning-making (ibid., 192), whereas ibasho means “one’s place to belong to” (ibid., 191) and relates to feeling at home, and therefore, oneself. Feeling lonely in Japan can lead to a sense of lost belonging and place—ibasho—that leads to a sense of loss of meaning, which becomes the cause for questioning one’s ikigai (ibid., 197). De-Silva suggests that because of political and social-structure changes, young Japanese struggle to find their *ibasho*, and this puts them at risk of suicide. With reference to the devastating earthquake of 2011, she writes that some people were displaced by their communities, and “by losing those environments that afforded their form of existence (their *ibasho*), they lost their way of life” (Osawa de-Silva, 2021, p. 175; parentheses ours).

In Jason Danely’s (2022) work on family caregivers in Japan and England, he discusses space in his description of how family carers learn to cope with the hardship of caregiving by engaging counter-worlds (ibid., 112). Counter-worlds are “other possible worlds or transcendent visions of possibility and aspiration” (ibid., 114). Often, they are spatially demarcated, and they may be reached through a state of mind, by hiking alone, engaging in spiritual practices, or joining support groups. What counter-worlds have in common is that they are means of navigating a complex caregiving story, and of “re-organizing the jumble of dark or disordered thoughts, feelings, and sensations” (ibid., 139). Like own-time spaces, counter-worlds exemplify how the dynamics of withdrawal or proximity may be understood as intrinsic to human relationality and temporality.

Unlike own-time spaces, counter-worlds seem to resemble aesthetic “rooms for reflection.” Whereas counter-worlds are described as “a state of mind,” and sometimes as involving a spiritual practice, and de-Silva writes about people who have lost the place where they belong, own-time spaces are often more or less deliberately cultivated, and they often involve embodied practices such as gardening, driving a truck, caring for horses, or photographing nature. We believe that searching for, and finding, ways to thrive may happen in various ways, and may be something humans experience only in glimpses, and in different ways. However, living with ADHD in a society that favors being on time and being productive is an invitation to determine how to live well. In this sense, finding and inhabiting own-time spaces is a kind of agentic practice that comes from experimenting with life with ADHD. Also, own-time spaces may be seen as expressions of the idea that time work (Flaherty, 2011) is both intra- and intersubjective. That is, the time work in which our interlocutors engage involves managing time in the world with others, but it also concerns their inner temporal experiences (Fuchs, 2013). And, meaning is key to understanding the effect of own-time spaces, and why own-time spaces “rhythm lives and bodies”—at least for a while.

## Conclusion

With this article, we have examined temporal dimensions of the lived experience of ADHD. We have contributed to an expanding literature suggesting that we see ADHD as a natureculture condition (Brinkman, 2024; Nielsen, 2017, 2020a, 2020b), thereby adding a layer to what Rasmussen and her colleagues describe elsewhere as a bio-chrono-social approach to ADHD. While neuropsychiatric literature describes time-blindness and time deficits in ADHD as pathologies, we, departing in a critical phenomenological understanding of the human subject, suggest that individuals living with ADHD are extra sensitive to time. Such perspectives are also forwarded in a growing literature on temporality and pathophysiological phenomenology on schizophrenia and depression (Fuchs, 2005, 2013) and bipolar disorder (Moskalewicz & Schwartz, 2020). By exploring ADHD through images, and the concepts of rhythm and lived time, we see how our interlocutors creatively expand psycho-educative strategies such as shielding, to responsively manage their ADHD symptoms.

The purpose of shielding as a therapeutic effort is to reduce stimuli and distractions, thereby creating calm and improving concentration. Shielding is often introduced and arranged by others for a child in school, for example. Own-time spaces work similarly, but expand the idea of shielding, because they work and are driven by dreams, the proximity and distance to other people, and thus relational sensitivity, and they draw on a lifetime perspective, as suggested in the imagistic thinking.

Although shielding is often introduced by others to improve concentration, and thus work performance, own-time spaces are established deliberately by people diagnosed to regulate their experience of ADHD, and to help them to thrive for a while. When Kenny went to school, he was seated behind a wooden divider to protect him from disturbing stimuli and improve his attention span. This did not work well for him at that time. However, as we now know, his truck, or his own-time space, serves as an excellent shield.

## Footnotes

1 Some interlocutors were diagnosed with ADHD (Attention Deficit Hyperactivity Disorder), others with ADD (Attention Deficit Disorder). Here, we do not distinguish between them, but use the term ADHD for both.

2 https://worldpopulationreview.com/country-rankings/adhd-rates-by-country

3 The numbers in the studies referred to here draw on data from the public sector of psychiatry and data that show how many people have prescriptions for ADHD medication. However, over the last years, a part of the assessments and treatments have been transferred to the private sector of psychiatry, and the number of the patients does not count here. Therefore, studies of prevalence in Denmark may be subject to uncertainty.

4 Timework is an agentic strategy carried out to modulate or manipulate the subjective experience of time, e.g., to make time pass faster when you are bored, or slower, if you wish to “stay in the moment” (see more in Flaherty, 2011)

5 https://www.addept.org/living-with-adult-add-adhd/sensory-overload-adults-adhd

6 https://adhd.dk/wp-content/uploads/2022/10/25-raad-og-redskaber-til-laerer-og-paedagoger.pdf We work with images as a methodological approach because images in these cases describe spaces and explain how and why spaces are experienced/´work´. Imagistic thinking as a method ensures curiosity and a state of openness to the world/Umwelt.

## References

[CR1] Bader, O., Bizzari, V., & Fuchs, T. (2022). Space, social perception, and mental disorders: Phenomenological and empirical approaches. *Psychopathology,**55*(3–4), 129–131. 10.1159/000524754 35580564 10.1159/000524754

[CR2] Barkley, R. A. (2005). *ADHD and the nature of selfcontrol*. The Guilford Press.

[CR3] Barkley, R. A. (2020). *Taking charge of ADHD, the complete, authoritative guide for parents*. The Guilford Press.

[CR4] Barkley, R. A., Murphy, K. R., & Bush, T. (2001). Time perception and reproduction in young adults with attention deficit hyperactivity disorder. *Neuropsychology,**15*(3), 351–360. 11499990 10.1037//0894-4105.15.3.351

[CR5] Berlant, L. (2022). *On the inconvenience of other people*. Duke University Press.

[CR6] Bernard, H. R. (2006). Interviewing: Unstructured and semistructured + fieldnotes: How to take them, code them manage them. *Research Methods in Anthropology,**21*, 387–412.

[CR7] Blum, K., Chen, A. L. C., Braverman, E. R., Comings, D. E., Chen, T. J., Arcuri, V., & Oscar-Berman, M. (2008). Attention-deficit-hyperactivity disorder and reward deficiency syndrome. *Neuropsychiatric Disease and Treatment.,**4*(5), 893–917. 19183781 10.2147/ndt.s2627 PMC2626918

[CR8] Brinkmann, S. (2024). What are mental disorders? Exploring the role of culture in the harmful dysfunction approach. *Integrative Psychological and Behavioral Science*. 10.1007/s12124-024-09837-9 10.1007/s12124-024-09837-9 PMC11638284 38565707

[CR9] Carceral, K. C., & Flaherty, M. (2021). *The cage of days. Time and temporal experience in prison*. Columbia University Press.

[CR10] Carelli, M. G., & Wiberg, B. (2012). Time out of mind: Temporal perspective in adults With ADHD. *Journal of Attention Disorders,**16*(6), 460466. 10.1177/1087054711398861 10.1177/1087054711398861 21490173

[CR11] Christensen, F. L. L. (2021). Synchronization and syncopation: Conceptualizing autism through rhythm. *Culture, Medicine and Psychiatry,**45*, 683–705. 10.1007/s11013-020-09698-y 33387160 10.1007/s11013-020-09698-y

[CR12] Conrad, P. (2007). Medicalization: Context, characteristics, and changes. *The medicalization of society. On the transformation of human conditions into treatable disorders. Part One: Concepts* (pp. 3–19). The Johns Hopkins University Press.

[CR13] Danely, J. (2022). *Fragile resonance. Caring for older family members in Japan and England*. Cornell University Press.

[CR14] Flaherty, M. (2011). *The textures of time*. Temple University Press.

[CR15] Fuchs, T. (2005). Implicit and explicit temporality. *Philosophy, Psychiatry and Psychology,**12*(3), 195–198. 10.1353/ppp.2006.0004

[CR16] Fuchs, T. (2013). Temporality and psychopathology. *Phenomenology and the Cognitive Sciences,**12*, 75–104. 10.1007/s11097-010-9189-4

[CR17] Fuchs, T. (2017). Collective Body Memories. In C. Durt, T. Fuchs, & C. Tewes (Eds.), *Embodiment, enaction, and culture. Investigating the constitution of the shared world* (pp. 333–349). The MIT Press.

[CR18] Fuchs, T. (2018). *Ecology of the brain*. Oxford University Press.

[CR100] Fuchs, T. (2020). Time, the body, and the other in phenomenology and psychopathology. In C. Tewes, G. Stanghellini (Eds.), *Time and body: Phenomenological and psychopathological approaches* (pp. 12–40). Cambridge University Press.

[CR19] Gillberg, C., Gillberg, I. C., Rasmussen, P., Kadesjö, B., Söderström, H., Råstam, M., Johnson, M., Rothenberger, A., & Niklasson, L. (2004). Co–existing disorders in ADHD – implications for diagnosis and intervention. *European Child and Adolescent Psychiatry,**13*(1), 80–92. 10.1007/s00787-004-1008-4 10.1007/s00787-004-1008-4 15322959

[CR20] Glavind, I. M. (2022). *Loss and belonging – Life with Alzheimer’s Disease in Denmark*. Ph.D. dissertation, department of Anthropology, University of Copenhagen (UPCH) and the Danish Alzheimer Association

[CR21] Goodwin, M. A. (2010). *On the other side of hyperactivity: An anthropology of ADHD*, University of California Berkeley. Unpublished PhD Thesis.

[CR22] Grinker, R. (2009). *Isabel’s world: autism and the making of a modern epidemic*. Icon.

[CR23] Husserl, E. (1964). Appendix XII Internal consciousness and the comprehension of lived experiences. *The phenomenology of internal time-consiousness* (pp. 175–181). Indiana University Press.

[CR24] Jackson, M. (2018). Surviving loss and remaking the world: Reflections on the Singular Universal in a West African Setting. *HAU Journal of Ethnographic Theory,**8*(1–2), 185–189.

[CR25] Laursen, C. B. (2023). *Gut trouble. Irritation, Experimentation, and Welfare in Denmark*. PhD-dissertation, Faculty of ARTS, Aarhus University.

[CR26] Lefebvre, H. (2022). *Rhythmanalysis space, time and everyday life*. Bloomsbury Academic.

[CR27] Madsen, K. B., Ersbøll, A. K., Olsen, J., Parner, E., & Obel, C. (2015). Geographic analysis of the variation in the incidence of ADHD in a country with free access to healthcare: A Danish cohort study. *International Journal of Health Geographics,**14*, 24. 10.1186/s12942-015-0018-4 26297014 10.1186/s12942-015-0018-4 PMC4546292

[CR28] Mattingly, C. (2014). *Moral laboratories. Family peril and the struggle for a good life*. University of California Press.

[CR29] Mattingly, C. (2019). Defrosting concepts, destabilizing doxa: Critical phenomenology and the perplexing particular. *Anthropological Theory,**19*, 415–439.

[CR30] Meinert, L. (2022). Together apart: Fence work in landscapes of relationality, old age, and care in the Ik mountains. In C. Mattingly & L. Grøn (Eds.), *Imagistic Care: Growing Old in a Precarious World red* (pp. 137–162). Fordham University Press.

[CR31] Moskalewicz, M., & Schwartz, M. A. (2020). Temporal experience in mania. *Phenomenology and the Cognitive Sciences,**19*, 291–304. 10.1007/s11097-018-9564-0

[CR32] Nielsen, M. (2017). ADHD and Temporality: A Desynchronized Way of Being in the World. *Medical Anthropology,**36*(3), 260–272. 10.1080/01459740.2016.1274750 28033486 10.1080/01459740.2016.1274750

[CR33] Nielsen, M. (2020a). ADHD and temporal experiences: Struggling for synchronization. In G. F. Michael, M. Lotte, & D. Line (Eds.), *Timework. Studies of temporal agency* (pp. 50–64). Berghan Books Timework.

[CR34] Nielsen, M. (2020b). *Experiences and explanations of ADHD. An ethnography of adults living with a diagnosis*. Routledge.

[CR35] Nielsen, M. (2021). At navigere mellem systemer med en ADHD-diagnose. *Jordens Folk,**56*(1), 52–61.

[CR101] Olsen, J. V., & Christensen, E. (2013). Inkluderede elever mistrives i folkeskolen. https://www.folkeskolen.dk/bornelivfolkeskolen-nr-18-2013-forskning/inkluderede-elever-mistrives-i-folkeskolen/782558

[CR36] O’Reilly, K. (2012). *Ethnographic methods*. Routledge.

[CR37] Osawa de-Silva, C. (2021). *What Lonelines can teach us. The anatomy of loneliness suicide, social connection, and the search for relational meaning in contemporary Japan* (pp. 190–219). University of California Press.

[CR38] Rapp, R. (2016). Big data, small kids: Medico-scientific, familial and advocacy visions of human brains. *BioSocieties,**11*(3), 296–316.

[CR39] Rasmussen, G. V., & Meinert, L. (2019). ADHD – En tidstypisk tidsforstyrrelse? Tidsarbejde Blandt Danske Familier der Lever Med ADHD. *Tidsskrift for Forskning i Sygdom Og Samfund,**30*, 199–217.

[CR40] Rasmussen, G. V., Meinert, L., & Flaherty, M. (2024). Time and ADHD in Danish Families: Mutual Affect Through Rhythm. *Medical Anthropology,**43*(7), 626–640. 10.1080/01459740.2024.2410244 39431902 10.1080/01459740.2024.2410244

[CR41] Rosa, H. (2014). *Alienation and Acceleration. Towards a Critical Theory of Late-Modern Temporality*. Århus University Press.

[CR42] Rosa, H. (2019). *Resonance. A Sociology of Our Relationship to the World*. Polity Press.

[CR43] Rose, N. (2007). *The politics of life itself. Biomedicine, power, and subjectivity in the Twenty-First Century*. Princeton University Press.

[CR44] Seeberg, J., & Christensen, F. L. (2017). Configuring the autism epidemic: Why are so few girls diagnosed? *Tidsskrift for Forskning i Sygdom Og Samfund,**26*, 127–144.

[CR45] Stevenson, L. (2014). *Life beside itself. Imagining care in the Canadian Arctic*. University of California Press.

[CR46] Stokker-Jensen, V. (2017). Performing autism through a layered account. Exploring the ambiguity of normative time and space. *Departures in Critical Qualitative Research,**6*(1), 72–94. 10.1525/dcqr.2017.6.1.72

[CR47] Thomsen, P. H. (2015). Børne- og Ungdomspsykiatriske diagnoser, *Diagnoser - perspektiver, kritik og diskussion*, red. Svend Brinkmann, and Anders Petersen, Forfatterne and Klim, Aarhus (129-154)

[CR48] Weinstein, N., Hansen, H., & Thuy-Vy, T. N. (2021). What time alone offers: narratives of solitude from adolescence to older adulthood. *In Frontiers in Psychology,**12*(714518), 1–15. 10.3389/fpsyg.2021.714518 10.3389/fpsyg.2021.714518 PMC8591032 34790144

[CR49] Williams, L., & Meinert, L. (2020). Repetition Work: Healing spirits and trauma in the churches of Northern Uganda. In L. Meinert & L. Dalsgaard (Eds.), *Timework. Studies of Temporal Agency* (pp. 31–49). Berghan Books.

[CR50] https://adhd.dk/wp-content/uploads/2022/10/25-raad-og-redskaber-til-laerer-og-paedagoger.pdf

[CR51] https://www.addept.org/living-with-adult-add-adhd/sensory-overload-adults-adhd

[CR52] https://www.folkeskolen.dk/borneliv-folkeskolen-nr-18-2013-forskning/inkluderede-elever-mistrives-i-folkeskolen/782558

[CR53] https://pro.medicin.dk/sygdomme/sygdom/318330

[CR54] https://worldpopulationreview.com/country-rankings/adhd-rates-by-country

